# Competition between members of the tribbles pseudokinase protein family shapes their interactions with mitogen activated protein kinase pathways

**DOI:** 10.1038/srep32667

**Published:** 2016-09-07

**Authors:** Hongtao Guan, Aban Shuaib, David Davila De Leon, Adrienn Angyal, Maria Salazar, Guillermo Velasco, Mike Holcombe, Steven K. Dower, Endre Kiss-Toth

**Affiliations:** 1 Department of Infection, Immunity & Cardiovascular Disease, University of Sheffield, Beech Hill road, Sheffield, S10 2RX, United Kingdom; 2 Department of Biochemistry and Molecular Biology I, School of Biology, Complutense University, Madrid, Spain; 3 Instituto de Investigación Sanitaria del Hospital Clínico San Carlos (IdISSC), Madrid, Spain; 4 Department of Computer Science, University of Sheffield, Beech Hill road, Sheffield, S10 2RX, United Kingdom; 5 Bio21 Biotechnology Institute, University of Melbourne, 30 Flemington Road, Parkville, Victoria, 3010, Australia; 6 CSL Limited, 45 Poplar Rd, Parkville, Victoria 3052, Australia

## Abstract

Spatio-temporal regulation of intracellular signalling networks is key to normal cellular physiology; dysregulation of which leads to disease. The family of three mammalian tribbles proteins has emerged as an important controller of signalling via regulating the activity of mitogen activated protein kinases (MAPK), the PI3-kinase induced signalling network and E3 ubiquitin ligases. However, the importance of potential redundancy in the action of tribbles and how the differences in affinities for the various binding partners may influence signalling control is currently unclear. We report that tribbles proteins can bind to an overlapping set of MAPK-kinases (MAPKK) in live cells and dictate the localisation of the complexes. Binding studies in transfected cells reveal common regulatory mechanisms and suggest that tribbles and MAPKs may interact with MAPKKs in a competitive manner. Computational modelling of the impact of tribbles on MAPK activation suggests a high sensitivity of this system to changes in tribbles levels, highlighting that these proteins are ideally placed to control the dynamics and balance of activation of concurrent signalling pathways.

Spatio-temporal control of intracellular signal transduction pathways is achieved by a range of mechanisms, including regulation of receptor expression, post-translational modifications of pathway components, expression of scaffolds that bring together critical components of the signalling pathway at specific locations, as well as the action of regulatory proteins, which can augment or inhibit pathway activation. However, most intracellular signalling proteins form families with high sequence homology and often share binding partners and targets. It is generally accepted that differences in binding affinities between homologous proteins and their partners are fundamentally important in shaping signalling responses. Yet, characterising these aspects of signalling control remain technically challenging. We have investigated the interaction between MAP kinase kinases (MAPKK) and the family of tribbles (TRIB) pseudokinases, using *in vitro* systems, to exemplify such signalling control mechanisms. These data provide a semi-quantitative insight into how altered relative expression of specific TRIB proteins may lead to the enrichment (or reduction) of distinct signalling complexes.

Tribbles (TRIB) form an evolutionally ancient family of pseudokinases^1^,^2^ and have been shown to interact with MAP kinase kinases (MAPKK)^3^,^4^, signalling molecules in the PI3K pathway^5^,^6^,^7^ and E3 ubiquitin ligases^8^,^9^,^10^, thereby regulating the activity of these pathways. It has been proposed that these interactions may be mechanistically important in the development of cancer^11^,^12^,^13^ as well as in the control of inflammation^14^,^15^,^16^.

Also, it has been shown that both TRIB1 and TRIB2 may be oncogenes in the development of acute myeloid leukaemia via similar mechanisms^17^, raising questions about potential functional redundancy between these proteins. Similarly, there is no consensus in the current literature about the oncogenic vs. tumour suppressor role of tribbles proteins^12^,^18^,^19^,^20^, suggesting that an important aspect of their activity may be context or cell type dependent. We believe that many of the currently conflicting published studies might be explained and reconciled if we understood the molecular basis of specificity and redundancy between tribbles proteins. Thus, we carried out a systematic analysis of TRIB/MAPKK interactions in mammalian cells and performed computational modelling to quantitatively assess the impact of tribbles on MAPK activation.

We report that tribbles and MAPKK proteins form inducible intermolecular complexes in live cells, mediated via the kinase-like domain of TRIBs and the N-terminus of MAPKKs. Estimation of the relative strength of TRIB-MKK4 binding revealed an up-to twenty fold differences between distinct tribbles, thereby suggesting that intracellular concentration (and trafficking) may be an important controller of TRIB action. In line with these experimental data, computational modelling of TRIB-mediated control of MAPK activation demonstrated that a ten-fold increase or decrease of TRIB concentration (or a similar change in TRIB/MAPKK affinity) is sufficient to switch the MAPK pathway between ON and OFF states.

Uncovering mechanistic details of signal transduction circuits is essential to understand how ubiquitously expressed proteins process a range of incoming signals to achieve cell-type and stimulus-specific cellular responses. Our current analysis provides experimental and computational evidence that the functional outcome from regulatory interactions between signalling kinases and TRIB proteins may be heavily influenced by the relative local abundance of both TRIBs and MAPKs.

## Results

### Tribbles subcellular localisation determines the intracellular distribution of trib/mapkk complexes

**Figure 1:**
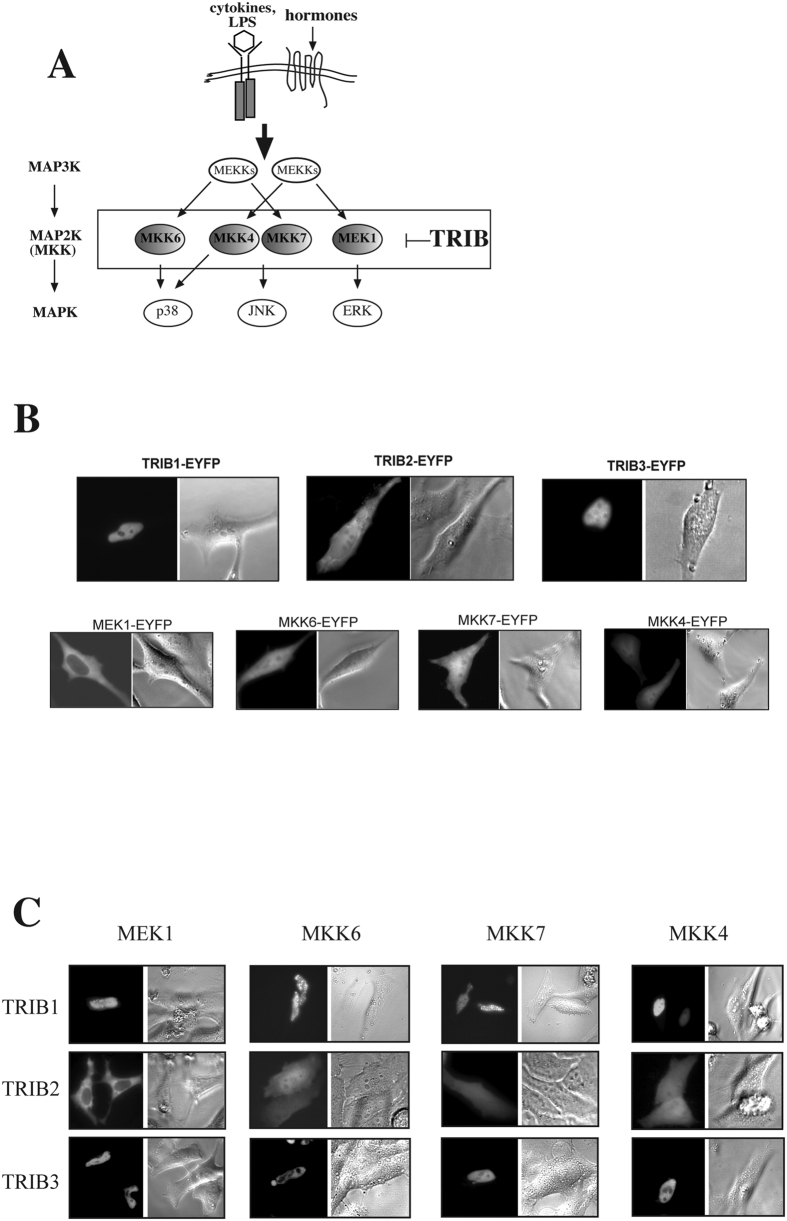
Members of the tribbles family interact with activators of all three groups of MAPKs. (**A**) A schematic diagram of TRIB mediated control of MAPK activation. (**B**) Plasmids expressing YFP tagged full length TRIBs or MKKs were transfected into HeLa cells. 24 hrs after transfection, intracellular TRIB expression profile was examined by fluorescent microscopy. Representative images are shown. (**C**) MKKs (MEK1, MKK4, 6, 7) and TRIB1-3 were fused to the V1 or V2 fragments of YFP, respectively, and co-transfected to HeLa cells. Localisation of fluorescent complexes was investigated by fluorescent microscopy. YFP fluorescent images and the corresponding phase contrast image are shown in panels B,C.

We and others have demonstrated previously that tribbles proteins exert their regulatory roles, at least in part, by shaping MAPK activation at the level of MAPKKs^13^,^21^,^22^,^23^ (Fig. 1A). However, we have also shown that tribbles action in inhibiting AP-1 activation is cell-type specific^22^, suggesting that the expression of additional, yet unknown binding partners or tissue specific modification of tribbles may also be key to their functioning. In order to gain a further mechanistic insight into tribbles action, here we undertook a systematic study, investigating the intracellular localisation of TRIB/MAPKK complexes. Interactions between the three tribbles and MEK1, MKK6, MKK4 and MKK7 (activators of ERK, p38 and JNK MAPKs, respectively) were tested in HeLa cells, where all three tribbles are endogenously expressed and both TRIB-1 and -3 were shown to inhibit MAPK activation^23^. In order to visualise the subcellular localisation of TRIB/MAPKK complexes, we used protein fragment complementation (PCA) assay, as described previously^15^,^24^. The technique is based on the ability of YFP to re-fold and form a functional fluorophore when two truncated versions of the protein (encoding the N- and C-terminal regions, respectively) come to close proximity to each other. YFP was split into two fragments and each was fused to a protein of interest (TRIB or MAPKK).

First, the distribution of the overexpressed MKKs or TRIBs as EYFP fusion proteins (MAPKK/TRIB-EYFP) was studied. In order to minimise overexpression artefacts, we have selected and analysed cells with low fluorescence levels, which have previously been shown to be close to physiological levels for inflammatory signalling molecules^25^. In agreement with previous findings, fluorescent microscopy results of TRIBs (1–3)-EYFP single transfected cells demonstrated that full-length TRIB1 and TRIB3 proteins are located in the nucleus, whereas TRIB2 protein is also expressed in the cytoplasm (Fig. 1B). Localisation of overexpressed MKKs (MEK-1, MKK6, MKK7 and -4) was examined in the same way. Full length MEK1 shows exclusive cytoplasmic expression, whereas the full length MKK6, MKK4 and MKK7 are all expressed over the whole cell (Fig. 1B). Broadly, these findings are in agreement with previous reports in the literature^26^,^27^.

Building on these data, the interaction between TRIBs and MKKs and the localisation of the TRIBs-MKKs complexes were examined. A systematic survey was carried out to detect MAPKK/TRIB complexes for MEK-1, MKK6, MKK7 and -4. Representative, high magnification images are shown in Fig. 1C, low power images and nuclear counter staining of selected complexes are also shown as additional controls (Suppl. Fig. 1A,B). All tested combinations of the TRIBs-MKKs pairs formed a detectable fluorescent complex; consistent with the hypothesis that tribbles are generic regulators of MAPKK activity. As additional controls, the interaction between endogenously expressed TRIB3 and MKKs was examined by co-immunoprecipitation (Suppl. Fig. 2). Complexes between all four MKKs and either TRIB1 or TRIB3 showed nuclear localisation, whereas complexes composed of the MKKs and TRIB2 showed both cytoplasmic and nuclear localisation, with the exception of the MEK1/TRIB2 complex, which is excluded from the nucleus (Fig. 1C).

### The tribbles kinase-like domain and the n-terminal region of mapkks are necessary for trib/mapkk interaction

Based on sequence homology, it is predicted that the structure of the MAPKKs and TRIBs are similar, in that both contain a kinase domain at the centre of the protein and an N- and a C-terminal domain with less understood function. The recently reported crystal structure for TRIB1 confirms these predictions^28^. However, the kinase domain in tribbles is thought to be catalytically inactive^29^, with the exception of TRIB2, which has recently been shown to carry a small auto-phosphorylating activity, *in vitro*^30^. In order to characterise the protein domains necessary for the formation and localisation of TRIB/MAPKK complexes, a series of deletion mutants were generated, expressing truncated versions of MKKs and Tribbles (Suppl. Table 1). We combined wild type TRIB with the deletion mutants of MKKs, or *vice versa* to perform the PCA experiments, similar to the above.

**Figure 2:**
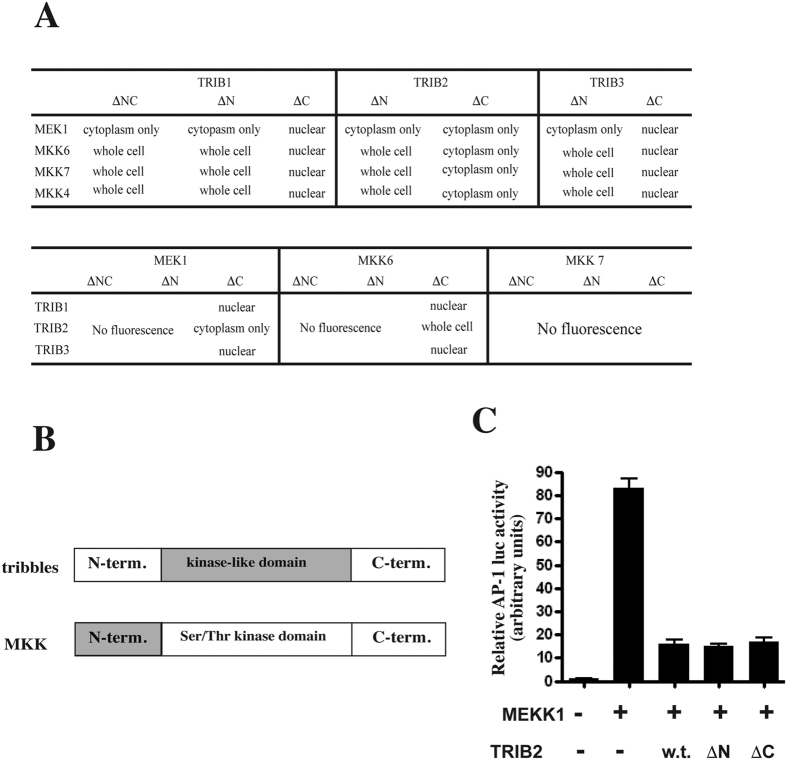
The central kinase-like domain of tribbles and the N-terminal region of MKKs are required for the formation of the TRIB/MAPKK complex. (**A**) PCA constructs, using full length or truncated versions of TRIBs or MKKs (as indicated in the Figure) were transfected to HeLa cells to map the regions of both binding partners required for the formation of TRIB/MAPKK complex. The intracellular localisation of complexes were examined by fluorescent microscopy and listed in the Figure. (**B**) Schematic representation of the regions (highlighted in grey) of both TRIB and MKKs, required for complex formation. (**C**) Full length (w.t.) or truncated versions of TRIB2 were expressed in HeLa cells and the ability of these TRIB proteins to inhibit MEKK1 driven activation of JNK- > AP-1, as measured by an AP-1 responsive luciferase reporter, was assessed. Bar graph shows Mean ± S.D. N = 4.

The results demonstrate that all truncated mutants of tribbles form complexes with wild type MKKs (Fig. 2A). However, altered cellular localisation was seen for TRIB1 and TRIB3/MAPKK complexes when N-terminal, or both N- and C-terminal tribbles domains were deleted. In particular, MEK1 - TRIB1/3ΔN complexes showed only cytoplasmic localisation, whilst MKK6/7 - TRIB1/3ΔN complexes were seen both in the cytoplasm and in the nucleus. These data are in line with the expression patterns of the various MKKs as shown on Fig. 1C (MEK1 cytoplasm only and MKK4, 6, 7 in the whole cell) and indicate that the N-termini of the TRIB1 and TRIB3 are important for localising the TRIB/MAPKK complexes in the nucleus. In contrast to TRIB1 and TRIB3, deleting the N-terminus of TRIB2 did not alter the localisation of MKKs-TRIB2 complexes (both cytoplasmic and nuclear). However, deleting the C-terminus of TRIB2 altered the localisation of the complex with MKK4/6/7, which were observed in the cytoplasm.

To complement the above experiments, the impact of deletion of N- or C-terminal domains of MKKs on TRIB/MAPKK complex formation was tested. For MKK7, both N- and C- termini were essential for interacting with tribbles as deletions of either domains eliminated the interaction with TRIB1-3. In contrast, only the N-terminal but not the C-terminal domains of MEK1 and MKK6 were essential for complex formation with TRIB1-3 (Fig. 2A). As a control, western blotting was undertaken to confirm the expression of the N-terminal truncated MAPKK proteins (Suppl. Fig. 3). As expected, these were expressed at levels similar to that of the full-length constructs. Therefore, we conclude that the loss of PCA signal is not due to lack of expression of the MAPKKΔN mutants.

In summary, we conclude that MKKs of all three MAPK pathways can interact with TRIBs and that TRIBs determine the localisation of the MAPKK/TRIB complex. Further, the N-termini of the MKKs and the central kinase-like domain of tribbles are indispensable for this interaction, with the exception of MKK7, where the C-terminus is also necessary (Fig. 2B). In line with our previous findings, N-termini of TRIB1 and TRIB3 but not of TRIB2 appear to be responsible for the nuclear localisation. However, we showed here that the C-terminal domain of TRIB2 may have a unique function as, unlike in other tribbles, its deletion leads to the exclusion of TRIB2/MAPKK complexes from the nucleus. Next, we confirmed that the kinase-like domain of TRIB2 not only is necessary for the interaction with MKKs, but it is sufficient for inhibiting MEKK1 driven AP-1 activation in HeLa cells (Fig. 2C). However, we note that some of these interactions may only take place in a cell type specific manner, as we observed TRIB2/MKK7 but not TRIB2/MKK4 complexes in monocytes previously, using PCA^15^.

### Formation of the tribbles/mapkk complex is inducible; trib and mapk proteins compete for mapkk binding

**Figure 3:**
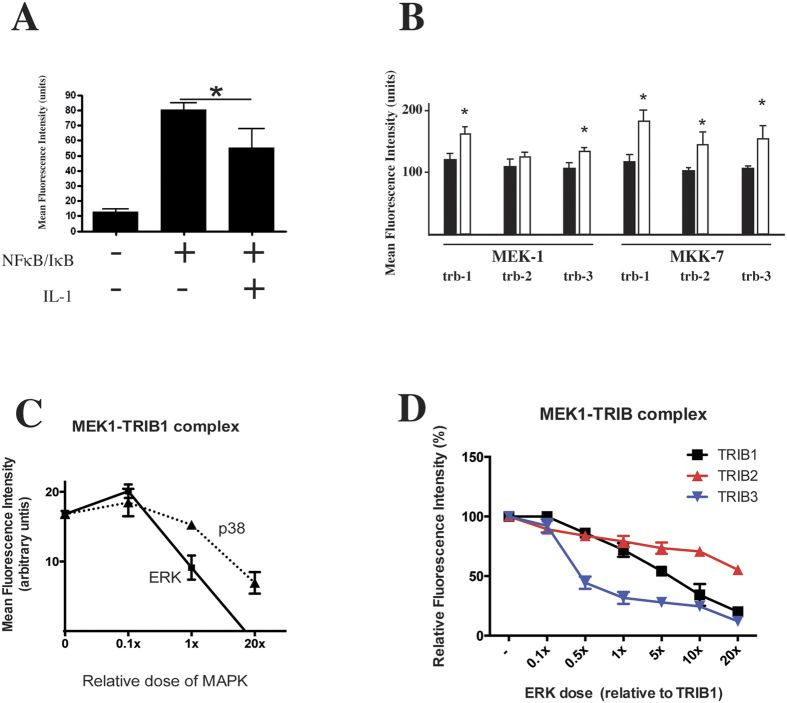
TRIB/MAPKK complex formation is induced by inflammatory activation and MAPKs compete with tribbles for MAPKK binding. (**A**) The ability of PCA to detect changes in protein complexes was verified by RelA-V1 and IκBα-V2 cotransfected HeLa cells, which were stimulated by IL-1α (0.1 nM, 60 min), 24 hrs after transfection. (*p < 0.05). (**B**) The impact of IL-1α (0.1 nM, 60 min) stimulation on MEK1-TRIB and MKK7-TRIB complexes was examined using flow cytometry, 24 hrs. post-transfection (*p < 0.05). (**C**) The relative intensity of the MEK1-TRIB1 PCA complex was analysed in the presence of an increasing dose of unlabelled ERK vs. p38 expression. N ≥ 3 (■: increasing ERK dose, ▲: increasing p38 dose). (**D**) The relative intensity of MEK1-TRIB PCA complexes were analysed in the presence of an increasing dose of unlabelled ERK (■: TRIB1, ▲: TRIB2, ▼: TRIB3).

Tribbles have previously been shown to regulate inflammatory signalling, one of the molecular mechanisms being via their interaction with MAPKKs^14^,^15^,^23^,^24^,^31^,^32^. However, the dynamics of TRIB/MAPKK complex formation and the relationship between TRIBs and MAPKs is currently unclear. Thus, we tested the impact of IL-1β stimulation on TRIB/MEK1 and TRIB/MK7 complexes, using the PCA assay, as above. As a validation of the PCA method’s suitability for intervention studies, we tested this system using a well characterised protein-protein interaction pair, NF-κB/RelA and IκB; the complex is disrupted by IL-1 mediated cellular activation. FACS analysis showed that RelA-v1 and IκBα-v2 formed a fluorescent complex, the intensity of which decreased significantly after 60 min of IL-1 stimulation, due to the degradation of IκBα (Fig. 3A). These results are in line with previous reports, where the dynamics of RelA/IκB complex was investigated using GFP-FRET^33^ and validate the use of PCA in investigations of the dynamics of formation/disruption of multi-protein complexes. Serum starved HeLa cells, transfected with MEK-1/TRIB or MKK7/TRIB PCA construct pairs were stimulated by a non-saturating dose of IL-1 (0.1 nM) for 60 min. The intensity of the PCA signal was quantified by flow cytometry. Data shown in Fig. 3B demonstrate that the formation of these complexes is induced by IL-1 treatment.

Next we wanted to characterise the relationship between TRIB and MAPK proteins, in the context of complex formation with MAPKKs. First, we used the MEK1/TRIB1 PCA complex and added increasing doses of untagged ERK (MEK1 binding MAPK) vs. p38 (non-MEK1 interacting MAPK) and shown that co-expression of equal dose of ERK vs. TRIB1 leads to a ~50% reduction in the intensity of the MEK1/TRIB1 PCA signal (Fig. 3C). In contrast, co-expression of p38 had no detectable impact at this dose, suggesting that there was a competitive relationship between TRIB and MAPK proteins for interacting with their MAPKK partner. Finally, we tested whether ERK disrupts MEK1/TRIB1-3 complexes with equal efficiency. Our data demonstrates that the MEK1/TRIB3 complex is most sensitive to co-expressed ERK (~50% of the MEK1/TRIB3 complex disrupted, in the presence of 0.5x the dose of TRIB3), followed by TRIB1 and TRIB2 (Fig. 3D). Interestingly, about 50% of the MEK1/TRIB2 PCA signal was preserved, even when 20 fold excess of ERK was co-expressed.

### Tribbles family members compete for binding with mapkks

**Figure 4:**
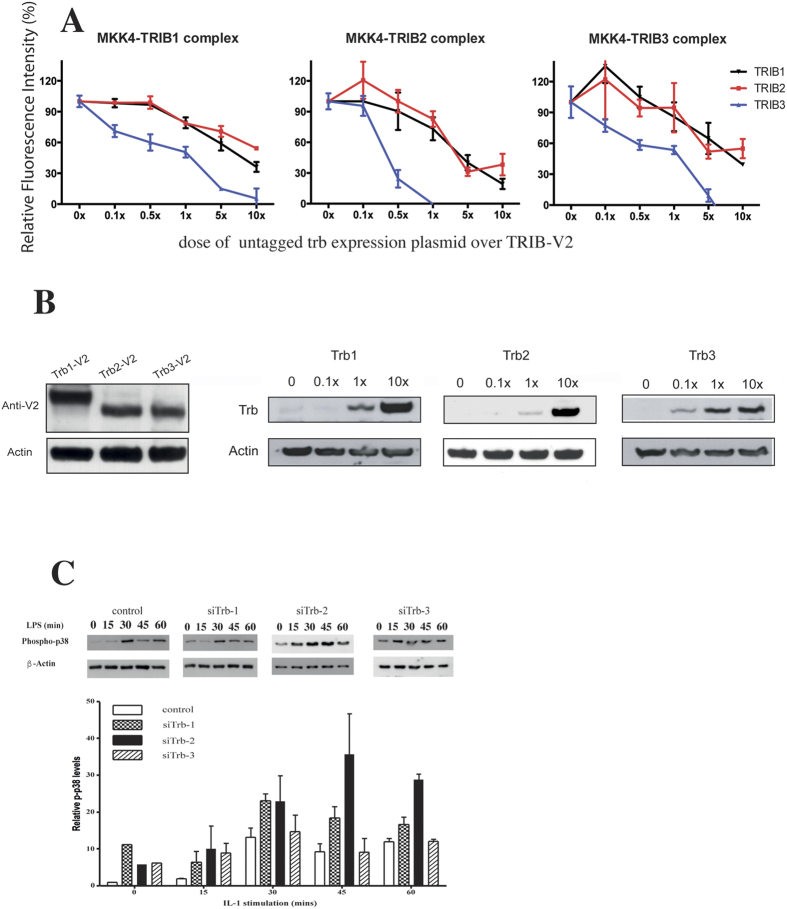
TRIB proteins compete with each other for MAPKK binding. (**A**) The relative intensity of the MKK4-TRIB PCA complexes were analysed in the presence of an increasing dose of unlabelled tribbles. N ≥ 3 (▼: TRIB1, ■: TRIB2, ▲: TRIB3) (**B**). Left panel: 400 ng expression plasmids, encoding for individual tribbles-V2 fusion proteins were transfected into HeLa cells and expression levels were detected by an anti-GFP western blot. Middle and right panels: Tribbles expression levels in HeLa cells increase in a dose dependent manner. Transfected doses of untagged tribbles, relative to the TRIB-PCA dose used in panel A are indicated above the individual panels. Representative western blots are shown (N = 3). (**C**) The differential impact of knockdown of specific tribbles on p38 MAPK activation was assessed in THP-1 cells. Cells transfected with non-targeting control or si-Trib constructs were stimulated by LPS for the stated length of time and the activation of p38 was detected by a phospho-p38 specific western blot. As loading control, the membrane was re-probed for β-actin. Upper panel: a representative result, Lower panel: quantitative assessment of p-p38 from three independent experiments. Data is expressed relative to the β-actin signal.

Since members of the tribbles family interact with an overlapping set of MAPKKs, the interactome between TRIBs-MAPKKs may be a continuously balanced, dynamic system, with tribbles turnover and expression levels being tightly regulated^34^,^35^,^36^. Given the data presented in Fig. 3C,D, we hypothesised that TRIB proteins may not only compete with MAPKs but they themselves bind to MAPKKs cross-competitively. However, in addition to differing expression levels, the binding affinities between distinct TRIBs and MAPKKs may also vary, resulting in an additional layer of complexity for pathway control. To address these questions in live cells, we have used the PCA assay as above and tested the binding of tribbles to MKK4. MKK4-V1 was co-transfected with a tribbles-V2 expression plasmid (Fig. 4A). We have then co-transfected an increasing dose of untagged tribbles expression plasmid, encoding TRIB-1, -2 or -3, respectively. Each MKK4/TRIB complex behaved similarly in that the level of fluorescent signal reduced as an increasing dose of untagged tribbles was co-expressed. In addition, TRIB-3 was substantially more potent in inhibiting fluorescent complex formation. Next, we confirmed that the three recombinant tribbles proteins are expressed at similar levels, when an identical dose of the expression plasmids is transfected, thus enabling comparison of relative binding affinity in live cells (Fig. 4B, left panel). In addition, transfection of an increasing dose of tribbles expression plasmid resulted in a parallel increase of tribbles expression levels (Fig. 4B). Data presented above led us to hypothesise that distinct TRIBs may have differential effects on signalling, in part due to their different “affinities” to their binding partners. This was addressed by using siRNA knockdown of individual tribbles in monocytes and showed that whilst knockdown of any of the three tribbles led to an increase in basal p-p38 levels, the impact on IL-1 induced p38 activation was distinct; siTrib-2 substantially augmented p-p38 levels, whilst siTrib-3 rendered monocyte p-p38 non-inducible by IL-1 (Fig. 4C).

**Figure 5:**
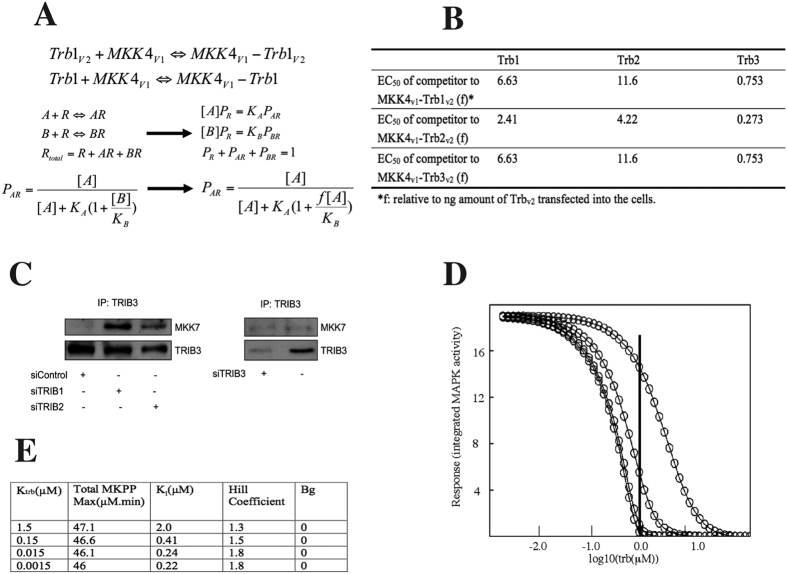
An ODE model to characterise the impact of tribbles mediated inhibition of MAPK activation. (**A**) A reversible competitive binding model was used to analyse FACS data presented on panel A by MLAB (Civilized Software Inc). (**B**) The EC_50_ values (f, fold to ng amount of TRIB-v2 transfected) indicate the amount of the untagged TRIB protein required to interrupt half of the MKK4V1-TRIBV2 complexes. (**C**) HepG2 cells were transfected with the indicated siRNA, followed by an IP for TRIB3. The level of MKK7 interacting with TRIB3 was measured by Western blot. (**D**) The effect of varying K_TRIB_ on the integrated response to the E1 pulse was calculated using the ODE system. TRIB concentrations were varied over ca 10^5^ range; time courses were simulated and integrated. (**E**) The integrated output dose responses were fitted with a cooperative inhibition model, the results are shown here.

The above competitive PCA assay demonstrated that TRIB-3 could titrate out the MKK4-TRIB binding most efficiently (Fig. 4A), indicating that the affinity of MKK4/TRIB-3 may be the highest among the three. Next, we quantitatively analysed the data by applying a reversible competition-binding model (Fig. 5A). Unlabelled TRIB protein binds MKK4-V1 as a competitor (“B” in equations, Fig. 5A), whilst fusion protein TRIB-V2 being the binding agent (“A”, Fig. 5A). In our experiment setting, equal amount (ng) of TRIB-V2 was added into each sample mix, and 0 to 10x competitor TRIB (relative to the TRIB-V2 dose) was added. We introduced an “f” coefficient in the equation so that [B] = f*[A] for fitting the optimal curves. This competition binding model assumes that 1) the interactions are reversible; 2) The binding agents already reached equilibrium when the FACS analysis was undertaken, 3) The degree of AR complex formation is proportional to the mean fluorescent intensity, and 4) Dose (μg) of plasmids transfected into the cells are proportional to the expression of the protein.

Based on these results, we conclude that of the three tribbles, TRIB-3 has ca 15 fold greater affinity to MKK4, compared to TRIB2, thus being the most efficient in competing with other tribbles in the formation of TRIB/MKK4 complex; with TRIB1 having an intermediate affinity (Fig. 5B).

Putting the above data together raises the possibility that altering the expression levels of specific tribbles may have an indirect impact on cell signalling and function by “liberating binding partners” for interaction with other TRIB proteins. We have explored this by knocking down TRIB1, TRIB2 or TRIB3 in HepG2 cells and measuring the level of interactions between TRIB3 and MKK7 under these conditions by co-immunoprecipitation (Fig. 5C and Suppl. Fig. 4). Our results demonstrate that reducing TRIB1 or TRIB2 levels leads to an enhanced TRIB3/MKK7 interaction, supporting the notion that TRIBs form a protein family which controls cell signalling in an integrated fashion.

### Model for inhibition of map kinase activity by tribbles proteins

In our original report on the characterisation of tribbles proteins^23^, we showed that when TRIB3 expression plasmid was titered in HeLa cells, all 3 MAPK cascades were maximally inhibited to the same extent (ca. 60–70%), but the mid points of the dose responses differed in the rank order p38 < JNK < ERK, and approximately at relative levels of 1:3:10, possibly reflecting differing affinities of TRIB3 for specific MKKs. This data fits well with results presented in Fig. 3D, demonstrating that ERK was most efficient in disrupting (and probably replacing) TRIB3 in its complex with MEK1. Data presented above (Fig. 4A) indicate that specific tribbles may also bind to the same MAPKK with varying affinity. Taken these results together, we wished to use a computational model and test the hypothesis that 10–20 fold change in the binding affinity of tribbles to its binding partner, or a similar change in TRIB expression levels is able to control the activation of MAPK cascades. Modelling was based on a 3 step map kinase cascade:


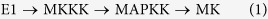


with each protein kinase requiring two sequential phosphorylation steps for activation. The properties of this type of model have been analysed elsewhere; for example taking Km values in the range 60–1500 nM for the individual protein kinase reactions yields ultrasensitive input/output characteristics for the system, with Hill coefficients for the MKPP output in the range of 3–6 and 50% maximal output as a function of E1 concentration ca 1000–10,000 lower than the Km values used for the individual kinase reactions^37^.

Our previous data suggest that tribbles proteins interact specifically with MKKs, and act as inhibitors of MAPK signalling^23^. We approached this problem by adopting the MAPK signalling model, originally described by Ferrell *et al*.^38^ and incorporated tribbles action into this system. Based on this (but *vide infra*), the full model for this analysis is shown in Suppl. Fig. 5, and the ODE system is presented in Appendix 1. Parameter values for enzyme concentrations and rate constants were taken from refs 37,38.

To model the transient responses typically observed in immune and inflammatory mediator action, in contrast to differentiation or cell cycle progression which are switch like and irreversible, we simulated a receptor generated signal rapidly attenuated for example by ligand-receptor complex internalisation and degradation, by replacing the E1 activator enzyme input concentration with the piecewise linear function:


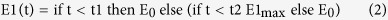


where t is time in minutes, such that t2 > t1, defining a square wave pulse at t1, width t2-t1 and amplitude E1_max_ above a constant background of E_0_. Using this input we tested a model based on that described by Ferrell and Machleder^38^, which incorporates an MK to MKKK positive feedback loop, and found, as expected, that this showed bistability in the response to the square wave input as either t2-t1 or E1_max_ were varied, and hence the system once ON, it did not return to baseline as t → infinity. This was not therefore useful for transient response modelling. Consequently, we did not incorporate positive feedback loops in the model.

We designated the specific locus of tribbles action, based on our and others findings. Specifically:

(a) Tribbles proteins act by binding to MKKs as above^15^,^23^,^24^.

(b) Tribbles proteins attenuate MK phosphorylation/activity as above^23^,^24^.

(c) Tribbles proteins do not attenuate MAPKK phosphorylation^24^.

(d) Tribbles are phosphoproteins and phosphorylation is induced by similar inputs to those that activate MKs^39^.

These observations suggest that a minimal plausible mechanism for tribbles action is competitive inhibition of MK phosphorylation by active MKKs; (MKKPP in the model used here). Thus either TRIBs do not bind to non-phospho- and mono-phospho-MAPKK, or if so then these complexes are also substrates for E1.MKKK, and the interaction has no functional impact on system behaviour. Finally, given (d) above, we examined whether requiring that tribbles be phosphorylated to be active, i.e. to bind to MKKPP, significantly modified TRIB effects when compared to a model where TRIB is intrinsically active (binding to MKKPP) without phosphorylation. We tested this experimentally by PCA and measured the effect of inhibiting MAPK activity on the capacity of all three TRIB proteins to bind to MKKs. IL1 treatment induced complex formation between TRIBs and MKKs (Fig. 3B). These data suggest that MK or elements downstream of MK act on the MAPKK step to potentiate MAPKK/TRIB interactions. To model this, given (d) we assumed that the tribbles kinase is MKPP and that it is the phosphorylation of TRIBs that activates binding to MKKPP, thus creating a negative feedback loop. While the evidence for this is circumstantial, it is a minimal assumption, avoiding arbitrarily introducing additional tribbles kinases into the system. In the parameter range close to that previously reported by Ferrel *et al*., sensitivity analysis over a wide range of values for TRIB interactions with both MKPP and MKKPP, suggested that introduction of the phosphorylation mediated negative feedback loop did not give rise to system behaviour qualitatively different from that produced by the simpler model in which unmodified TRIB was the active form. A credible biological explanation for this observation may be that TRIB phosphorylation impacts on its intracellular distribution, thus only the phospho form may be co-localised with MKPP. Nevertheless, given earlier reports in the literature and the data in Figs 2 and 3, we used the more complex model for further analysis.

To examine the predicted quantitative impact of varying TRIB/MAPKK affinity on the inhibition dose response curve (Fig. 5D), K_TRIB_ was set at 1.5, 0.15, 0.015 and 0.0015 μM and initial TRIB (t = 0) varied from 100 to 0.001 μM, with all other initials and constants fixed (Fig. 4G). Again, a square wave E1 input was used. The simulations show that once K_TRIB_ < 0.1 K_m_, little further effect on the inhibition dose response is found. However in the 0.1 to 10 range of the K_TRIB_/K_m_ ratio, and at TRIB concentrations in the range 1 to 10 μM, selective inhibition of parallel MAPK pathways would be predicted to occur. Thus, as the arrow in Fig. 3F indicates, when TRIB is 1 μM, if K_TRIB_ for 3 different MKKs were in the ratio 0.1:1:10 – the relative pathway activities would be 0:0.4:1. Further, the relative mid points in the inhibition dose response curves are 1:2:8.5, similar to those observed experimentally (ref. 23 and Fig. 4). Thus, a 100-fold range of K_TRIB_ could produce selective responses from the 3 MAPK cascades even if they all were intrinsically equally responsive to an input, provided that TRIB concentration was in slight (<1.5–2x) excess over MAPKK.

## Discussion

Since the original description of tribbles as regulators of morphogenesis and several signalling pathways, this family of proteins have increasingly been recognised as an important controller of cellular processes; dysregulation of tribbles expression/function has been implicated in a number of diseases, including hyperlipidaemia and myocardial infarction (reviewed in refs 14,40,41). One significant aspect of tribbles action, still ill defined, is the specificity and redundancy between the three mammalian proteins. Whilst TRIB1 and TRIB2 but not TRIB3 have been suggested to play a role in the development of acute myeloid leukemia (AML)^42^, TRIB2 and TRIB3 have both been shown to interact with PI3K and Akt^6^,^12^,^19^. On the other hand, the action of tribbles has been proposed to be cell type specific, the molecular basis for which is still not well understood. Whilst it is tempting to speculate that differences in intracellular localisation of distinct tribbles, their affinity to their binding partners as well as their expression levels may vary in a cell type specific manner, thus ultimately leading to specificity in tribbles action, most of these parameters have not been assessed systematically.

In the current study, we attempted to carry out a comprehensive study to ask the question: how does tribbles binding to MAPKK translate into specific signalling responses? We addressed this question by investigating the localisation of TRIB/MAPKK complexes, the requirement of specific protein domains for these interactions, the control of complex formation and by studying the impact of tribbles on MAPK activation, using a combined experimental and computational approach.

In line with previous data^43^, we show in here that TRIB1 and TRIB3 are expressed in the nucleus, whilst TRIB2 is predominantly expressed in the cytoplasm. In agreement with historical reports^26^,^27^, expression of MKKs, however, (with the exception of MEK1) appears to be less specific with respect to cellular compartmentalisation. The importance of TRIB localisation is reflected in the specific intracellular localisation pattern of their complexes with MKKs (Fig. 1C), raising the possibility that distinct pools of MKKs may interact with specific tribbles, and these complexes may be coupled to responses to specific stimuli. Whilst we have no direct evidence to support this hypothesis, it is tempting to speculate that distinct intracellular pools of signalling molecules, such as MKKs, with specific signalling functions may exist. Given these considerations, the relative “affinity” between TRIBs and MKK4 measured in live cells in our PCA system may not be comparable directly to values that would be obtained in a system using purified proteins, bearing in mind that most proteins with cells are likely not in free solution. Rather, it reflects a more complex “measure” of interactions by taking the physical availability of binding partners into account. However, it is clear from our data (Fig. 3D) that intracellular localisation may not be the sole determinant of the effectiveness by which distinct tribbles “compete” for MKKs. There is a notable difference in relative binding “affinity” between TRIB1 and TRIB3, despite the fact that both of these proteins appear to be expressed in the nuclear compartment (Fig. 1B).

The technique we chose to use here to investigate the binding between tribbles and MKKs has some limitations. Once a fluorescent complex forms, it “locks” the binding partners together, thus quantification of dynamic disruption of complexes with reversible binding is not possible. However, induction of complex formation can be followed (Fig. 3B). It has also been demonstrated previously that that dynamics of protein complexes can be studied in the limited cases of formation of new complexes, prevention of formation of new complexes or changes in localization of constitutive complexes with the YFP PCA^44^,^45^. Additionally, degradation mediated disruption of complexes may also be detected via the loss of fluorescent signal, as we show for the NFκB/IκBα complex (Fig. 3A). These limitations were taken into consideration in the design and interpretation of experiments presented here by investigating the impact of “competition” between PCA tagged and untagged TRIBs 24 hrs post-transfection, when the system reached a steady state. Thus, prevention of complex formation, rather than an impact on existing complexes were measured in the experiments, results of which were used for the modelling.

Finally, we and others have shown previously that expression level of specific tribbles varies substantially in a tissue^23^ and differentiation^34^ specific manner. This provides experimental support for the hypothesis that tribbles levels may be important in regulating qualitative, dynamic aspects of signalling. Using our experimental data, we have simulated the impact of altered tribbles concentration/affinity on the activation of MAPKs, utilising an ODE model initially developed by Ferrell and his colleagues to analyse MAPK signalling in oocytes^37^,^38^. Output from this analysis predicts that a ten-fold change in tribbles “affinity” (or in expression levels), a range observed experimentally, is sufficient to alter signalling outcome in the MAPK system.

However, ODE models have clear limitations when used for modelling of signalling events. For instance, they treat the cell as a homogeneous solution. Whilst they are undoubtedly useful to probe the impact of altered availability of certain components (in this case tribbles) on signal propagation, quantitative conclusions from these studies need to be interpreted with some caution. It is well established that MAPK activation (and other signalling events) take place in a specialised intracellular microenvironment, in scaffolded multi-protein complexes. Our data suggest that TRIBs may only interact with a small proportion of MAPKKs. For instance, whilst most MEK1 is seen in the cytoplasm (Fig. 1B), the TRIB1/MEK1 *complex* is located in the nucleus. This may explain (at least in part), why TRIBs that are often expressed at relatively low levels are still able to regulate signalling effectively. In these scaffolded complexes, local concentrations of components may be vastly different from those that is usually measured by most traditional biochemical analyses, using whole cell lysates. Consequently, the ratios between the components of the ODE modelled pathway are best interpreted to reflect local protein concentrations required within signalling complexes, rather than cell-wide expression levels.

Lately, novel modelling approaches are being developed and utilised to study signalling, that have the ability to account for spatio-temporal aspects of signal propagation. We have recently published the first agent-based model for MAPK signalling and demonstrated that the presence of scaffolded complexes may explain the ultrasensitivity of MAPK activation, as observed experimentally^46^. Current work in our group includes the incorporation of TRIB proteins into this framework to further our understanding of the molecular mechanisms of TRIB mediated signalling control.

Of note, our modelling analysis here have focussed on the inhibitory action of TRIB proteins in signalling. However, we have previously reported a concentration dependent, bi-phasic regulatory role for these proteins^23^ and potentiation of TRIB mediated potentiation of MEK1/ERK signalling has also been reported^13^,^47^. Whilst it is possible that complex alterations between specific MAPK complexes due to changes in specific TRIB levels could explain these results, such hypothesis is yet to be tested, both experimentally and theoretically, via computational models.

Whilst the experiments reported here have focussed on the interactions between TRIBs and MKKs, we believe that the same paradigm could also be applied for their molecular interactions with other partners, such as members of the PI3K signalling network and E3 Ub ligases. Therefore, we believe that lessons learned in this system and our main conclusions are generalizable and thus provide a holistic model that contributes a better understanding of how TRIB pseudokinases function at the molecular level.

## Materials and Methods

### Protein-fragment Complementation Assay (PCA)

In order to examine the physical interactions between tribbles and MAP kinase kinases, MEK1, MKK6, MKK4 and MKK7 in live cells, we used the yellow fluorescent protein (YFP) based protein fragment complementation assay (PCA). This strategy was developed and previously described by Michnick and his colleagues^48^,^49^. We have validated this approach to study interactions between tribbles-1 and MKKs^15^,^24^,^50^. The Venus variant of YFP was used in this study, since it provides a greater signal than EYFP (Venus variant YFP fragments were termed as V1 and V2 throughout this study). Full length GFP or YFP fragments were fused to the C-terminus of TRIB and MAPKK proteins, respectively.

### Plasmids

The plasmids encoding for full length TRIB and MAPKK in fusion with GFP or the Venus fragment have been generated as described before^15^,^24^.

### Cell culture and transfection

HeLa, THP1 and HepG2 cells were purchased from ATCC and maintained according to the supplier’s recommendations. siRNA SmartPools against human TRIBs -1, -2 and -3, and non-targeting siRNA (siNC) were purchased from Dharmacon and used as recommended by the manufacturer. Polyfect (Qiagen, Crawley, UK) was used for transfection into HeLa and HepG2 cells, according to the manufacturer’s instructions. THP-1 cells were transfected using Nucleofector (Amaxa).

### Fluorescence microscopy

Fluorescence images were taken by a Leica DMI4000B Inverted microscope (Leica Geosystems Ltd., Milton Keynes, UK). Representative fluorescent and phase contrast images were taken at 40x objective lens.

### FACS analysis

24 h after transfection, the cells were collected and flow cytometric analysis (FACS) was performed on a FACSCalibure (Becton Dickson, USA) following the general guidelines from the manufacturer. Data were analysed using Cell Quest Pro software (Becton Dickinson, USA). The fluorescent intensity values were normalised by subtracting the background fluorescent values for mock-transfected cells. 20,000 cells were analysed for each sample.

### Western blotting

Anti MKK4 and MKK7 antibodies was purchased from Cell Signalling Technology (Beverly, USA). Rabbit polyclonal GFP (ab290, 1/2500 dilution) and anti-TRIB3 (ab50516) were purchased from Abcam (Cambridge, UK). Anti-actin (c-11) antibody was purchased from Santa Cruz Biotechnology, Inc.

### Immunoprecipitation

HepG2 cells were lysed in HEPES lysis buffer (40 mM HEPES, pH 7.5; 120 mM NaCl, 1 mM EDTA, 10 mM sodium pyrophosphate, 10 mM sodium glycerophosphate, 50 mM NaFl, 0.5 mM sodium orthovanadate; 0.3% CHAPS). Lysate (1–4 mg) was precleared by incubating with 5–20 μl of protein G–Sepharose conjugated to pre-immune IgG. The lysate extracts were then incubated with 5–20 μl of protein G–Sepharose conjugated to 5–20 μg of the anti-TRIB3 antibody (ab50516) or pre-immune IgG. TRIB3 antibody was covalently conjugated to protein G–Sepharose using dimethyl pimelimidate. Immunoprecipitations were carried out for 1 h at 4 °C on a rotatory wheel. The immunoprecipitates were washed 4 times with HEPES lysis buffer, followed by 2 washes with HEPES kinase buffer. The immunoprecipitates were resuspended in 30 μl of sample buffer (not containing 2-mercaptoethanol) and filtered through a 0.22-μm Spin-X filter, and 2-mercaptoethanol was added to a concentration of 1% (vol/vol). Samples were subjected to electrophoresis and immunoblot analysis.

### Luciferase assay

The dual luciferase reporter assay system (Promega, Madison, WI) was used following the manufacturer’s instructions. Normalised relative luciferase activity was calculated as a ratio of firefly to Renilla luciferase activity for each sample.

### IL-1α treatment

IL-1α was a kind gift from Immunex Inc., USA.

### Statistical Analysis

All the experiments were repeated a minimum of three times, all graphs show a mean ± S.D. One-way ANOVA (followed by Tukey’s Multiple Comparison Test) or two-way ANOVA (followed by Bonferroni post-tests) were used to assess statistical significance, as appropriate.

## Additional Information

**How to cite this article**: Guan, H. *et al*. Competition between members of the tribbles pseudokinase protein family shapes their interactions with mitogen activated protein kinase pathways. *Sci. Rep.* **6**, 32667; doi: 10.1038/srep32667 (2016).

## Supplementary Material

Supplementary Information
