# Supplementary material for: Competition between members of the tribbles pseudokinase protein family shapes their interactions with mitogen activated protein kinase pathways
Source: Sci Rep. 2016 Sep 7;6:32667. doi: 10.1038/srep32667 (PMC5013389; doi:10.1038/srep32667)
Supplement: Supplementary Information [file srep32667-s1.pdf]

Supplementary information for:

Competition between members of the tribbles pseudokinase protein family shapes their interactions with mitogen activated protein kinase pathways

Hongtao Guan, Aban Shuaib, David Davila De Leon, Adrienn Angyal, Maria Salazar, Guillermo Velasco, Mike Holcombe, Steven K. Dower, and Endre Kiss-Toth

LEGENDS FOR SUPPLEMENTARY FIGURES:

Supplementary Figure 1: **Intracellular localisation of TRIB/MEK1 complexes.**
PCA constructs, encoding for full length of TRIBs and MEK1 were transfected to HeLa cells, as indicated in the figure. 24 hrs after transfection, intracellular YFP expression profile was examined by fluorescent microscopy.
A: Representative fields from 10x images. **B**: High power images from cells transfected as in A. Cell nuclei were visualised by counter staining with DAPI.

Supplementary Figure 2: **Interaction between endogenous TRIB3 and MKKs**
HepG2 cell lysates were used to detect interaction between endogenously expressed TRIB3 and MAPKK proteins. TRIB3 was immunoprecipitated, and subjected to gel electrophoresis and transferred to a nitrocellulose membrane. Co-precipitation of MKK4 and MKK7 with TRIB3 was then detected. As control, the membrane was also developed using the anti-TRIB3 Ab (bottom panel).

Supplementary Figure 3: **Expression of truncated (ΔN) MAPKK proteins**
Expression plasmids, encoding for truncated MKKs (fused to the V1 tag) were transfected into HeLa cells and their expression was detected by western blotting, using an anti-GFP antibody

Supplementary Figure 4: **The effect of siRNA knockdown on TRIB and MKK7 expression levels**
HepG2 cells were transiently transfected by siRNAs against specific TRIBs, as stated and the level of TRIB mRNA knockdown (4A) and the impact of siTRIB treatment on MKK7 (4B) was assessed by qRT-PCR and Western blotting, respectively.

Supplementary Figure 5: **The ODE model to characterise the impact of tribbles mediated inhibition of MAPK activation**
For each reaction, **ai** is the association rate and **di** is the dissociation rate. For the enzyme reactions, **ki** is the catalytic rate. Species for which time dependence is solved are in solid rectangles; species held fixed are in dashed rectangles. The individual reactions are numbered in both schemes. The tribbles "module" is outlined with a dotted line.

Supplementary Table 1: Amino-acid residues encoded by expression plasmids used in our PCA assays, encoding for full length or truncated forms of MKKs and tribbles.

Appendix 1: Equations of the ODE model for tribbles interaction with MAPK cascades

Supplementary figure 1: Intracellular localisation of trb/MEK1 PCA complexes

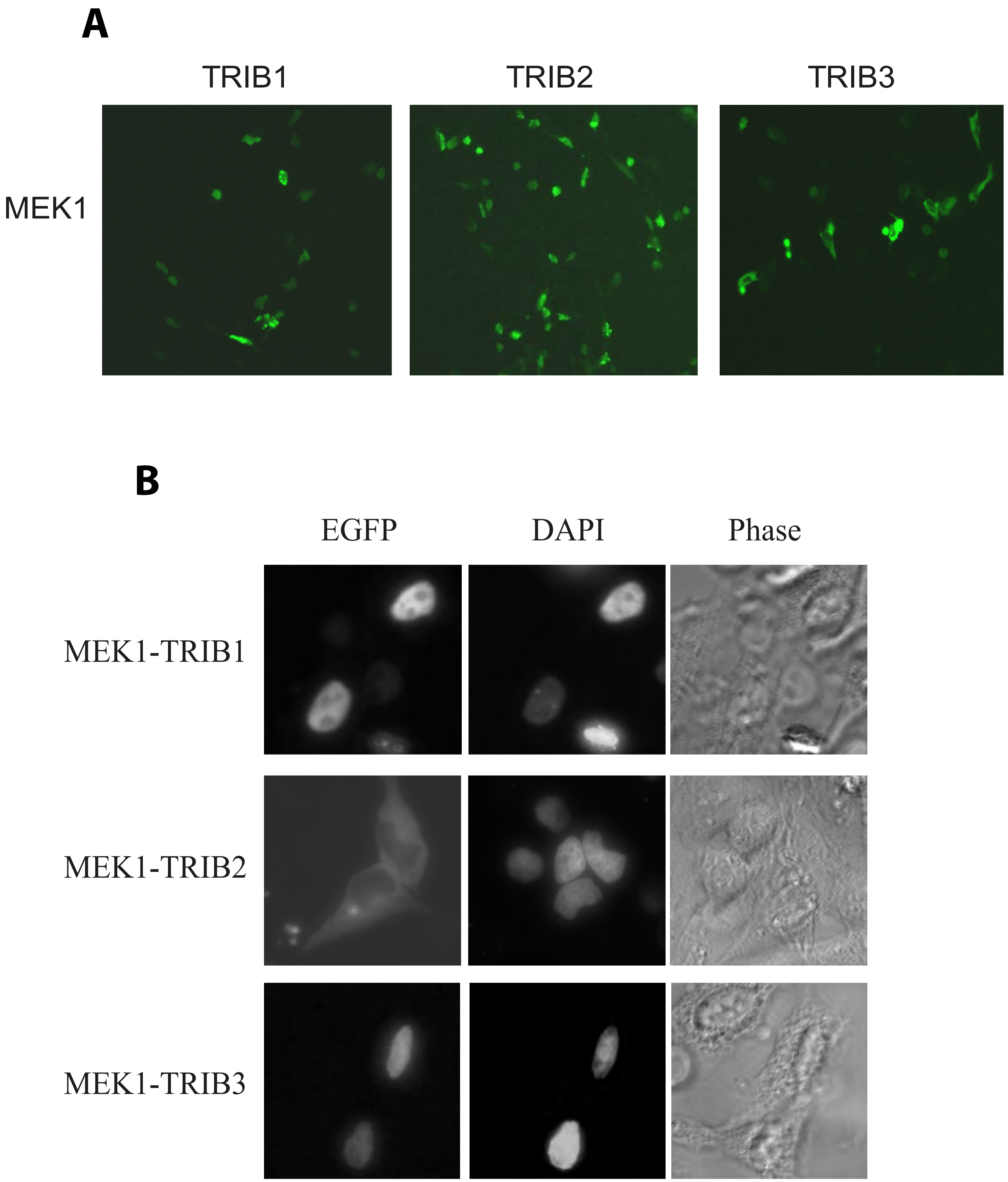

Supplementary figure 2: Interaction between endogenous TRIB3 and MKKs

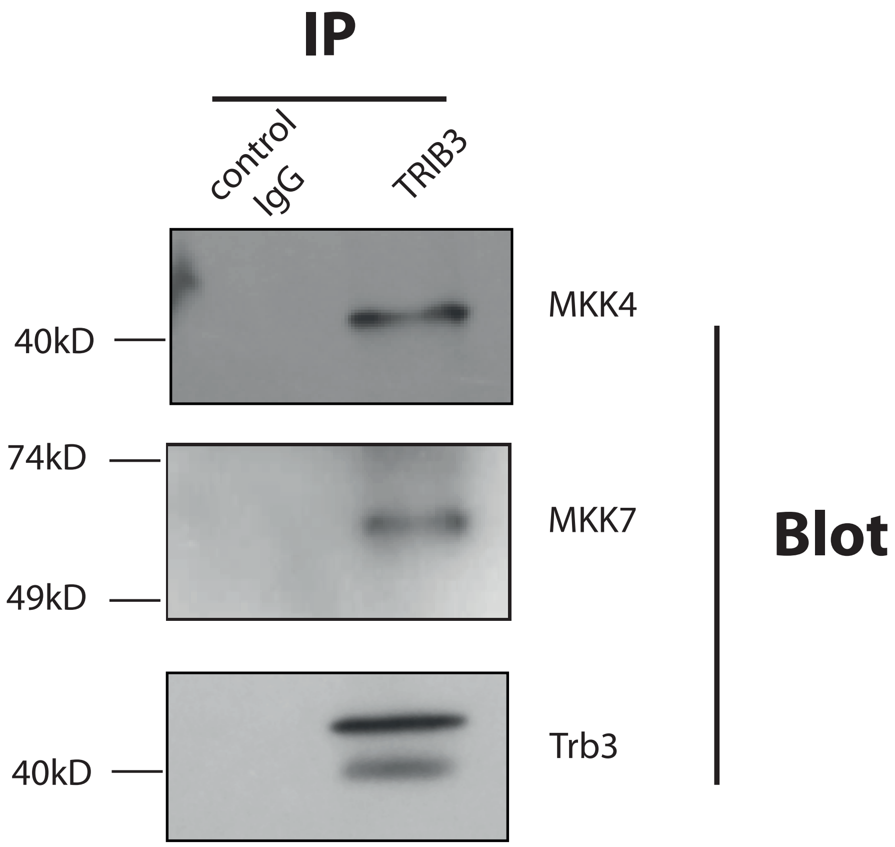

Supplementary figure 3:

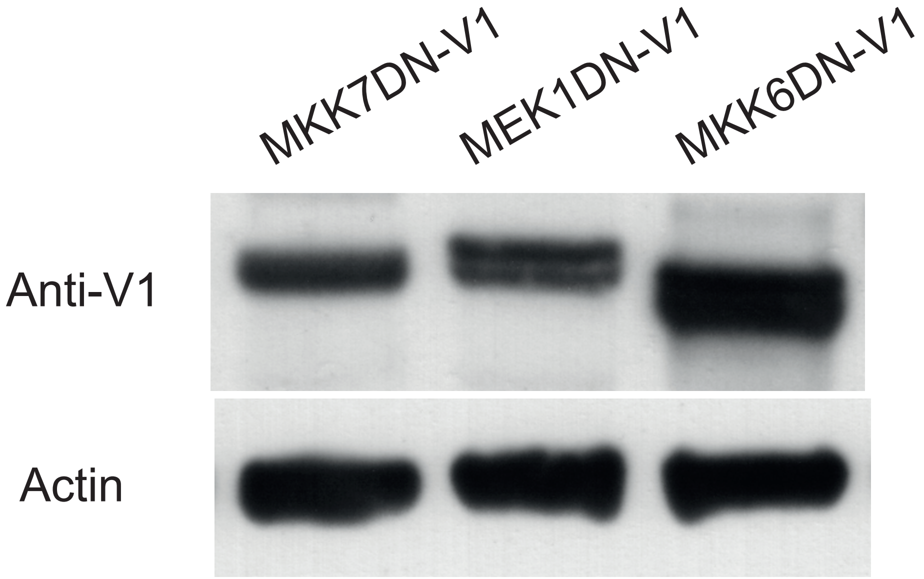

Supplementary Figure 4

A

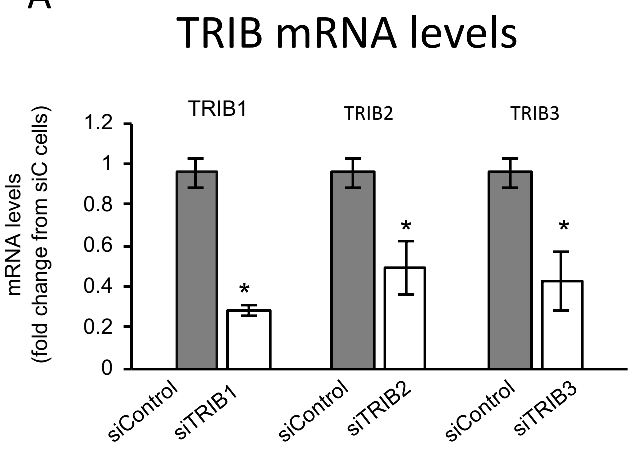

B

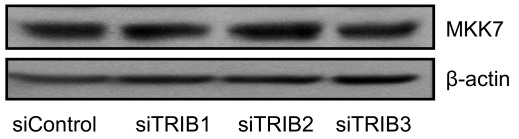

Supplementary Figure 5

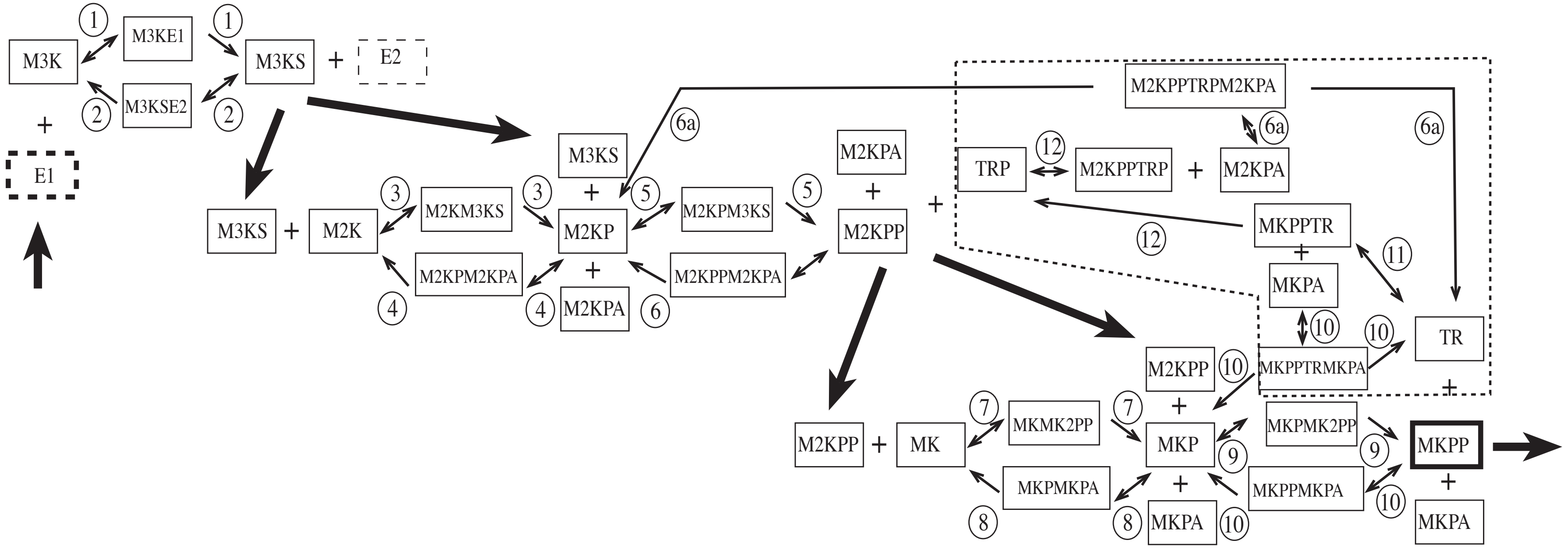


```
M3K+E1          <=> M3KE1         ->  M3KS +E1        :  rate constants a1, d1, k1   (1
M3KS+E2         <=> M3KSE2        ->  M3K + E2        :  rate constants a2, d2, k2   (2
M2K+M3KS        <=> M2KM3KS       ->  M2KP + M3KS     :  rate constants a3, d3, k3   (3
M2KP+M2KPA      <=> M2KPM2KPA     ->  M2K+M2KPA       :  rate constants a4, d4, k4   (4
M2KP+M3KS       <=> M2KPM3KS      ->  M2KPP +M3KS     :  rate constants a5, d5, k5   (5
M2KPP+M2KPA     <=> M2KPPM2KPA    ->  M2KP+M2KPA      :  rate constants a6, d6, k6   (6
M2KPPTRP+M2KPA  <=> M2KPPTRPM2KPA->  M2KP+M2KPA+TR   :  rate constants a6, d6, k6   (6a
MK+M2KPP        <=> MKM2KPP       ->  M2KPP +MKP      :  rate constants a7, d7, k7   (7
MKP+MKPA        <=> MKPMKPA       ->  MK+MKPA         :  rate constants a8, d8, k8   (8
MKPPTR+MKPA     <=> MKPPTRMKPA    ->  MKP+MKPA+TR     :  rate constants a8, d8, k8   (8a
MKP+M2KPP       <=> MKPM2KPP      ->  M2KPP +MKPP     :  rate constants a9, d9, k9   (9
MKPP+MKPA       <=> MKPPMKPA      ->  MKP+MKPA        :  rate constants a10,d10,k10  (10
MKPP+TR         <=> MKPPTR        ->  MKPP+TRP        :  rate constants a11,d11,k11  (11
M2KPP+TRP       <=> M2KPPTRP                          :  rate constants a12,d12      (12
```

For each equilibrium ai is the association rate and di the dissociation for the enzyme reactions ki is the cataytic rate species for which time dependence is solved are in solid rectangles , species held fixed are in dashed rectangles. The individual reactions are numbered in both schemes. The tribbles "module" is outlined with a dotted line.

Suppl. Table 1

MKK-V1 constructs				TRIB-V2 constructs			
Wild type	ΔN	ΔC	ΔNC	Wild type	ΔN	ΔC	ΔNC
MEK 1 (aa 1-393)	**(aa 68-393)**	**(aa 1-362)**	**(aa 68-362)**	TRIB-1 (aa 1-372)	**(aa 92-372)**	**(aa 1-338)**	**(aa 91-338)**
MKK 6 (aa 1-334)	**(aa 53-334)**	**(aa 1-307)**	**(aa 53-307)**	TRIB-2 (aa 1-343)	**(aa 61-343)**	**(aa 1-307)**	
MKK4 (aa 1-400)	**(aa 102-400)**		**(aa 102-334)**	TRIB-3 (aa 1-358)	**(aa 67-358)**	**(aa 1-315)**	
MKK7 (aa 1-419)	**(aa 127-419)**	**(aa 127-380)**	**(aa 127-380)**				

Appendix 1 – Model for Tribbles interaction with Map kinase cascade

From Figure 2, the system contains the following 28 species of which 26 are explicit in the ODE system :-
(E1, E2,)M3K, M3KS, M3KE1, M3KSE2, M2KM3KS, M2KPM3KS, M2K, M2KP, M2KPP, M2KPA, M2KPM2KPA, M2KPPM2KPA, MKM2KPP, MKPM2KPP, MK, MKP, MKPP, MKPMKPA, MKPPMKPA, MKPA, MKPPTR, TR, TRP, M2KPPTRP,M2KPPTRPM2KPA, MKPPTRMKPA
2 are defined algebraically:-
E1 is described by a piecewise defined linear function that defines a square wave pulse,
E2 is treated as constant.

The differential equation system is

```
e1(t)                = if t<t1 then e0 else (if t<t2 then ee else e0)          (1
m3k't(t)             = -a1*m3k*e1(t)+d1*m3ke1+k2*m3kse2                          (2
m3ke1't(t)           = a1*m3k*e1(t) -(d1+k1)*m3ke1                               (3
m3ks't(t)            = -a2*m3ks*e2+d2*m3kse2+k1*m3ke1+(k3+d3)*m2km3ks-
                       a3*m3ks*m2k+(k5+d5)*m2kpm3ks-a5*m2kp*m3ks                 (4
m3kse2't(t)          = a2*m3ks*e2-(d2+k2)*m3kse2                                 (5
m2k't(t)             = -a3*m2k*m3ks+d3*m2km3ks+k4*m2kpm2kpa                      (6
m2km3ks't(t)         = a3*m2k*m3ks-(d3+k3)*m2km3ks                               (7
m2kp't(t)            = -a4*m2kp*m2kpa+d4*m2kpm2kpa+k3*m2km3ks+
                       k6*m2kppm2kpa+ d5*m2kpm3ksa5*m2kp*m3ks+
                       k6*m2kpptrpm2kpa                                          (8
m2kpm2kpa't(t)       = a4*m2kp*m2kpa -(d4+k4)*m2kpm2kpa                          (9
m2kpm3ks't(t)        = a5*m2kp*m3ks-(d5+k5)*m2kpm3ks                             (10
m2kpp't(t)           = k5*m2kpm3ks-a6*m2kpp*m2kpa+d6*m2kppm2kpa-
                       a7*m2kpp*mk+(d7+k7)*mkm2kpp-
                       a9*mkp*m2kpp+(d9+k9)*mkpm2kpp-
                       a11*trp*m2kpp+d11*m2kpptrp                                (11
m2kppm2kpa't(t)      = a6*m2kpp*m2kpa-(d6+k6)*m2kppm2kpa                         (12
m2kpptrpm2kpa't(t) = a6*m2kpptrp*m2kpa-(d6+k6)*m2kpptrpm2kpa                     (13
mk't(t)              = -a7*mk*m2kpp+d7*mkm2kpp+k8*mkpmkpa                        (14
mkm2kpp't(t )        = a7*mk*m2kpp-(d7+k7)*mkm2kpp                               (15
mkp't(t)             = k7*mkm2kpp-a8*mkp*mkpa+d8*mkpmkpa-
                       a9*mkp*m2kpp+d9*mkpm2kpp +
                       k10*(mkppmkpa+mkpptrmkpa)                                 (16
mkpmkpa't(t)         = a8*mkp*mkpa-(d8+k8)*mkpmkpa                               (17
mkpm2kpp't(t)        = a9*mkp*m2kpp - (d9+k9)*mkpm2kpp                           (18
mkpp't(t)            = -a10*mkpp*mkpa + d10*mkppmkpa+k9*mkpm2kpp-
                       a12*mkpp*tr+(d12+k12)*mkpptr                              (19
mkppmkpa't(t)        = a10*mkpp*mkpa - (d10+k10)*mkppmkpa                        (20
m2kpa't(t)           = -a6*m2kpp*m2kpa+(d6+k6)*m2kppm2kpa- a4*m2kp*m2kpa
                       +(d4+k4)*m2kpm2kpa-a6*m2kpptrp*m2kpa
                       +(d6+k6)*m2kpptrpm2kpa                                    (21
```

```
mkpa't(t)          = -a8*mkp*mkpa+(d8+k8)*mkpmkpa-a10*mkpp*mkpa
                      +(d10+k10)*mkppmkpa-a10*mkpptr*mkpa
                      +(d10+k10)*mkpptrmkpa                          (22
tr't(t)            = k6*m2kpptrpm2kpa -a12*mkpp*tr+d12*mkpptr
                      +d10*mkpptrmkpa                                (23
m2kpptrp't(t)      = a11*trp*m2kpp-d11*m2kpptrp-a6*m2kpptrp*m2kpa
                      +d6*m2kpptrpm2kpa                              (24
trp't(t)           = k12*mkpptr-a11*trp*m2kpp+d11*m2kpptrp           (25
mkpptr't(t)        = a12*mkpp*tr-(d12+k12)*mkpptr-a10*mkpptr*mkpa
                      +d10*mkpptrmkpa                                (26
mkpptrmkpa't(t)    = a10*mkpptr*mkpa- (d10+k10)*mkpptrmkpa           (27
```

There is a corresponding set of conservation equations implicit in the ODE system

```
m3k0    =  m3k+m3ks+m3ke1+m3kse2+m3ksm2k+m3ksm2kp                    (1
e10     =  e1+m3ke1                                                  (2
e20     =  e2+m3kse2                                                 (3
m2k0    = m2k+m2kp+m2kpp+m2km3ks+m2kpm3ks+m2kpm2kpa+
           m2kppm2kpa+ m2kppmk+m2kppmkp+m2kpptrp+m2kpptrpm2kpa       (4
m2kpa0  = m2kpa+m2kpm2kpa+m2kppm2kpa+m2kpptrpm2kpa                   (5
mk0     = mk+mkp+mkpp+mkmk2pp+mkpm2kpp+mkpmkpa+mkppmkpa+
           mkpptr+mkpptrmkpa                                         (6
mkpa0   = mkpa+mkpmkpa+mkppmkpa+mkpptrmkpa
tr0     = tr+mkpptr+trp+m2kpptrp+mkpptrmkpa+mk2pptrpm2kpa            (7
```

Numerical simulations were run with the following rate constant values such that Km for all reactions is 0.3 μM. The dissociation rate constant d11 is varied to change the Kd of phosphotribbles (trp) for active map kinase kinase(M2KKPP) .

a1 to a12 = 1000 $\mu M^{-1}.min^{-1}$
d1 to d12 = 150 min^{-1}
k1 to k12 = 150 min^{-1}

all initial concentrations were set to zero with the exception of the following

Input signal parameters:-

e0 = 2 pM	basal level of E1 activity
ee = varied from 1 pM to 1 mM	maximal E1 activity
t1 = 210 min	start for the activation signal – by which time the system reaches steady state
t2 = 220 min	end of activation signal
e2 = 0.3 nM	

Pathway component concentrations

m3k (t=0) = 3 nM
m2k (t=0) = 1.2 μM
mk (t=0) = 1.2 μM
m2kpa(t=0) = 0.3 nM
mkpa (t=0) = 0.6 μM – based on comparison between model and data in figure 6B
tr (t=0) = 1- 2 μM or varied over a wide range as described in the results section

the values for the component concentrations are taken from the literature.
